# Association of Baseline Systemic Immune‐Inflammation Index With Severe Sleep Disturbance in Patients With Neuropathic Pain: A Prospective Longitudinal Observational Cohort Study

**DOI:** 10.1155/prm/7604030

**Published:** 2026-08-29

**Authors:** Jialei Zhang, Xiaoling Zhang, Jing Li, Kai Cao, Jie Wu

**Affiliations:** ^1^ Department of Pain Treatment, Changzhi People’s Hospital Affiliated to Changzhi Medical College, Changzhi, China; ^2^ Department of Basic Medical Sciences, Shanxi Medical University, Taiyuan, China, sxmu.edu.cn; ^3^ Department of Oncology, Changzhi People’s Hospital Affiliated to Changzhi Medical College, Changzhi, China

**Keywords:** melatonin, neuropathic pain, Pittsburgh Sleep Quality Index, pulsed radiofrequency, sleep quality, systemic immune-inflammation index

## Abstract

**Background:**

Patients with neuropathic pain frequently experience impaired sleep quality. The systemic immune‐inflammation index (SII) is a readily available blood‐based inflammatory marker, but its association with sleep disturbance in neuropathic pain patients remains insufficiently defined.

**Methods:**

This single‐center prospective longitudinal observational cohort study enrolled 135 patients aged 40–65 years with neuropathic pain confirmed by the DN4 questionnaire, disease duration of 6–12 months, and pain inadequately controlled by conventional medication. Baseline severe sleep disturbance was defined using a prespecified severity‐based threshold of PSQI > 10. Fasting blood and first‐morning spot urine samples were collected at baseline and at the 1‐month follow‐up after PRF. SII was calculated as platelet count *x* neutrophil count/lymphocyte count. Native urinary melatonin concentration was measured by ELISA and reported as an unadjusted spot urine concentration. The primary analysis evaluated the association between baseline SII and severe sleep‐disturbance status; post‐PRF changes were interpreted as within‐subject temporal changes because no control group was included.

**Results:**

At baseline, 73 of 135 patients met the prespecified criterion for severe sleep disturbance. Compared to the nonsevere group, the severe group had higher baseline SII (803.03 ± 111.45 vs. 604.21 ± 164.29, *p* < 0.001) and lower urinary melatonin concentration (8.32 ± 2.17 vs. 9.61 ± 2.72, *p* = 0.006). At the 1‐month follow‐up, severe sleep‐disturbance status was present in 9 patients (McNemar test, *p* < 0.001). SII decreased, and urinary melatonin concentration increased in both baseline severity groups (all within‐group *p* < 0.001). In a limited multivariable logistic regression model adjusted for available baseline covariates, baseline SII was associated with severe sleep‐disturbance status (adjusted OR per 1‐unit increase: 1.011; 95% CI: 1.007–1.014; *p* < 0.001). Exploratory ROC analysis yielded an apparent in‐sample AUC of 0.833.

**Conclusions:**

Higher baseline SII was associated with severe sleep‐disturbance status in this mixed neuropathic pain cohort. The observed longitudinal changes after PRF should be interpreted cautiously as within‐subject temporal changes rather than PRF‐specific effects. The findings are exploratory and do not establish causality, a direct inflammatory mechanism, or validated predictive performance.

**Trial Registration:** Chinese Registry of Clinical Trials: ChiCTR2500103587

## 1. Introduction

Sleep is essential for maintaining cardiovascular, immune, cognitive, and emotional functions. Sleep disturbance can involve insufficient sleep duration, impaired sleep continuity, altered sleep architecture, and poor subjective sleep quality, all of which may negatively affect quality of life and clinical outcomes [1].

Chronic pain is defined as pain that persists or recurs for more than 3 months and represents a complex sensory and emotional experience [2]. Neuropathic pain is caused by a lesion or disease of the somatosensory nervous system and may arise from a broad range of peripheral or central nervous system conditions [3, 4]. Sleep problems are highly relevant in neuropathic pain because pain can disrupt sleep, while poor sleep may also amplify pain perception and inflammatory activity [5–7].

The systemic immune‐inflammation index (SII), calculated from peripheral platelet, neutrophil, and lymphocyte counts, has been used as a composite marker of systemic inflammatory and immune status [8, 9]. Compared to cytokine panels, SII is inexpensive, routinely available, and easily calculated from standard blood counts. However, its relationship with severe sleep disturbance in neuropathic pain patients has not been fully explored.

Melatonin is closely related to circadian regulation and sleep–wake physiology. In urine, 6‐sulfatoxymelatonin is generally regarded as the major metabolite used to estimate nocturnal melatonin production [10, 11]. In the present study, however, native urinary melatonin rather than urinary 6‐sulfatoxymelatonin (aMT6 s) was measured; therefore, the urinary results should be interpreted cautiously as unadjusted first‐morning spot urinary melatonin concentrations.

The present study aimed to evaluate whether baseline SII was associated with severe sleep‐disturbance status in a mixed neuropathic pain cohort and to describe within‐subject temporal changes in SII, urinary melatonin concentration, and severe sleep‐disturbance status at 1 month after PRF. Because this study did not include a standard care, sham procedure, or untreated control group, post‐PRF changes were not interpreted as PRF‐specific therapeutic effects.

## 2. Experimental Procedures

### 2.1. Study Design

This was a single‐center prospective longitudinal observational cohort study. Eligible patients with neuropathic pain were enrolled before PRF and underwent repeated assessments at two predefined time points: baseline and 1 month after PRF. The baseline component evaluated the association between SII and severe sleep‐disturbance status, whereas the longitudinal component described within‐subject temporal changes in SII, urinary melatonin concentration, and severe sleep‐disturbance status after PRF.

No standard‐care, sham‐procedure, or untreated control group was included. Therefore, the study was not designed to determine PRF‐specific efficacy or whether observed post‐PRF changes exceeded the natural disease course, placebo or contextual effects, regression to the mean, medication‐related effects, or pain‐relief‐related effects.

The study was approved by the Medical Ethics Committee of Changzhi People’s Hospital Affiliated to Changzhi Medical College and was registered with the Chinese Clinical Trial Registry. Written informed consent was obtained from all participants.

### 2.2. Participant Selection and Eligibility Criteria

Patients aged 40–65 years with suspected neuropathic pain who visited the Pain Department of Changzhi People’s Hospital Affiliated to Changzhi Medical College between January 2025 and October 2025 were screened. Neuropathic pain was confirmed using the Douleur Neuropathique 4 (DN4) questions questionnaire, with a score of ≥ 4 considered compatible with neuropathic pain [12].

The inclusion criteria were as follows: age 40–65 years, neuropathic pain duration of 6–12 months, pain inadequately controlled by conventional medication before PRF, no clinically significant liver or kidney dysfunction, ability to complete the required assessments, and provision of written informed consent. The 6–12‐month disease‐duration restriction was used to reduce heterogeneity related to disease chronicity.

The exclusion criteria were as follows: known infectious disease or autoimmune disease; recurrent neuropathic pain requiring repeated visits; history of traumatic brain injury, cranial surgery, or cerebral infarction; severe drug allergy; other chronic pain conditions; long‐term analgesic use before enrollment; participation in another clinical trial; and inability to cooperate with diagnosis, treatment, or follow‐up assessments.

After PRF, routine analgesic therapy was not scheduled. Rescue gabapentin 100 mg was allowed only when patient‐reported NRS was > 4. However, longitudinal NRS trajectories and rescue‐medication exposure, including timing, frequency, cumulative dose, and detailed pain trajectories, were not recorded in a sufficiently standardized format for adjusted modeling.

### 2.3. Observational Indicators

Baseline information included age, sex, height, weight, BMI, smoking history, alcohol use, selected comorbidities, and neuropathic pain etiology. Etiologies included headache‐related neuropathic pain, trigeminal neuralgia, herpes zoster–related neuralgia, intercostal neuralgia, peripheral neuropathy, and lumbar disc–related radicular pain.

Fasting venous blood was collected at baseline and at the 1‐month follow‐up. Platelet count, neutrophil count, and lymphocyte count were measured using a hematology analyzer. SII was calculated using the following formula: SII = platelet count *x* neutrophil count/lymphocyte count.

### 2.4. Urinary Melatonin Assay and Sample Handling

First‐morning spot urine was collected at baseline and at the 1‐month follow‐up before breakfast. Native urinary melatonin concentration, rather than aMT6 s, was measured using a commercial ELISA kit (Mlbio, Shanghai, China; Cat. No. ml024033) according to the manufacturer’s instructions. Urinary creatinine was not measured, and creatinine normalization was not performed. Therefore, the urinary melatonin results are reported as unadjusted first‐morning spot urinary melatonin concentrations rather than creatinine‐adjusted aMT6 s estimates of total nocturnal melatonin secretion.

### 2.5. Neuropathic Pain and Sleep Quality Assessment

The DN4 questionnaire was used to identify neuropathic pain. The questionnaire consists of symptom descriptors and clinical examination items, with a total score ranging from 0 to 10. A score of ≥ 4 was considered compatible with neuropathic pain [12].

Sleep quality was assessed using the Pittsburgh Sleep Quality Index (PSQI), a widely used self‐rated questionnaire evaluating sleep quality over the preceding month, and the conventional PSQI cutoff of > 5 is commonly used to indicate poor sleep quality [13]. In the present study, PSQI > 10 was used as a prespecified severity‐based threshold to identify patients with more pronounced sleep impairment in this pain‐clinic cohort, based on prior chronic pain literature [14]. Therefore, PSQI > 10 was not interpreted as a general diagnostic threshold for sleep disorders. Patients with PSQI > 10 were classified into the severe group, and those with PSQI ≤ 10 were classified into the nonsevere group.

### 2.6. Pulsed Radiofrequency Therapy

After the patient enters the operating room, electrocardiogram (ECG) monitoring, noninvasive blood pressure monitoring, and pulse oximetry are connected. An intravenous access is established. Under ultrasound guidance, the target nerve is selected, and the puncture route is clarified. Local anesthesia is administered with 1% lidocaine. A 20G radiofrequency puncture cannula needle is inserted along the marked route under imaging guidance. Once the puncture is successful, the radiofrequency treatment device is connected. Motor (0.5–2 mA, 2 Hz) and sensory (0.5–1 mA, 50 Hz) tests are performed to confirm the positioning. The treatment mode is then set for pulsed radiofrequency therapy at 42°C, 2 Hz, 45 V, and 200 s. After the procedure is completed, the patient is observed for 20 min. If there are no significant adverse reactions, the patient is returned to the ward.

### 2.7. Statistical Analysis

Data analysis was performed using SPSS Statistics Version 23.0 and Python‐based statistical verification. All analyses were based on the final complete‐case analysis set, including patients who completed both baseline and 1‐month follow‐up assessments and had available SII, urinary melatonin concentration, and severe sleep‐disturbance status data. No statistical imputation was performed.

Sample size considerations were originally based on an a priori conservative planning assumption of a moderate association (|*r*| = 0.30), a two‐sided alpha level of 0.05%, and 80% power. This estimate was used for feasibility planning before data analysis and was not intended to predict the final observed effect size or to support a definitive prediction model.

In the revised analysis, the primary sleep‐related outcome was severe sleep‐disturbance status, defined as PSQI > 10 versus PSQI ≤ 10. Therefore, the final analyses were interpreted as exploratory. The complete‐case cohort included 135 patients, with 73 patients in the severe group and 62 in the nonsevere group. Given that the multivariable logistic regression model included three available baseline covariates (sex, BMI, and baseline SII), the number of outcome events was considered sufficient for a limited exploratory adjustment model. However, the sample size was not sufficient for comprehensive confounder adjustment, formal prediction‐model development, or validated cutoff estimation.

The Shapiro–Wilk test was used to assess the distribution of continuous variables before selecting parametric or nonparametric tests. For independent between‐group comparisons, normality was assessed within severity groups. For paired baseline‐to‐follow‐up comparisons, the distribution of within‐subject difference scores was assessed. Normally distributed continuous variables were described as mean ± standard deviation and compared using independent‐samples *t*‐tests or paired‐samples *t*‐tests. Non‐normally distributed variables were compared using the Mann–Whitney *U* test or Wilcoxon signed‐rank test. Categorical variables were expressed as numbers and percentages and analyzed using chi‐square tests, continuity‐corrected chi‐square tests, Fisher’s exact tests, or McNemar’s test, as appropriate.

Because the longitudinal component included only two repeated time points, repeated‐measures ANOVA was not used for within‐subject comparisons, and assumptions such as sphericity were not applicable.

For exploratory logistic regression, candidate variables were limited to baseline covariates available in the analyzable dataset. Variables with *p* < 0.10 in univariate analyses among available baseline covariates were considered for entry into the multivariable model. The model was interpreted as a limited exploratory adjustment model rather than a fully specified clinical prediction or causal model. No formal mediation analysis was prespecified or performed. ROC analysis was used to describe the apparent in‐sample discriminative ability of baseline SII for severe sleep‐disturbance status. The cutoff value was exploratory and was not internally or externally validated. A two‐sided *p* < 0.05 was considered statistically significant.

## 3. Results

### 3.1. Comparison of General Conditions

**FIGURE 1:**
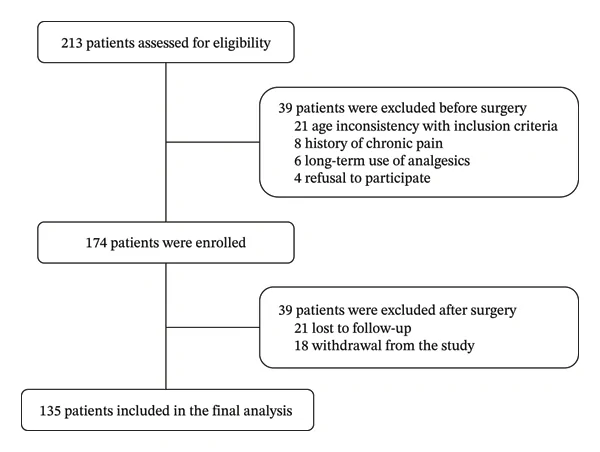
Flow diagram of participant screening, enrollment, follow‐up, and final analysis.

From January to October 2025, a total of 213 patients with neuropathic pain were admitted to the pain department of our hospital. Among them, 174 met the inclusion criteria, and ultimately, 135 patients completed the entire trial. Patients were divided into two groups based on the presence of sleep disorders before treatment: the sleep disorder group (SD group) and the nonsleep disorder group (Non‐SD group). There were no significant differences between the two groups in terms of gender, age, BMI, comorbidities, smoking history, and alcohol consumption history (*p* > 0.05). (Figure 1; Table 1).

**TABLE 1:** Demographic and baseline characteristics.

	All patients *n* = 135	Severe group *n* = 73	Nonsevere group *n* = 62	*p* value
Age, years	57.36 ± 7.64	56.89 ± 7.92	57.92 ± 7.69	0.410
Male, *n* (%)	61 (45.2)	31 (42.5)	30 (48.4)	0.491
BMI, kg/m^2^	23.87 ± 2.65	23.61 ± 2.81	24.17 ± 2.44	0.087
Smoking history, *n* (%)	51 (37.8)	30 (41.1)	21 (33.9)	0.494
Alcohol use, *n* (%)	28 (20.7)	17 (23.3)	11 (17.7)	0.563
Comorbidity, *n* (%)				
Hypertension	56 (41.5)	33 (45.2)	23 (37.1)	0.437
Coronary heart disease	17 (12.6)	11 (15.1)	6 (9.7)	0.496
Chronic lung diseases	4 (3.0)	3 (4.1)	1 (1.6)	0.731

*Note:* Severe group was defined as PSQI > 10 at baseline. *p* values for continuous variables were based on Mann–Whitney *U* tests when normality assumptions were not met. The etiology comparison used a chi‐square test and should be interpreted cautiously because some categories were sparse.

### 3.2. Sample Size and Outcome Clarification

The original sample size consideration was based on a conservative planning assumption of a moderate association (|*r*| = 0.30). After revision, the primary sleep‐related outcome was defined as severe sleep‐disturbance status rather than continuous PSQI score. Therefore, the previously reported continuous PSQI correlation coefficient was not used to support the main conclusions. The final complete‐case cohort included 135 patients, including 73 with severe sleep disturbance and 62 without severe sleep disturbance, which was considered adequate for limited exploratory association analyses but not for definitive prediction‐model development or causal inference.

### 3.3. Severe Sleep‐Disturbance Status at Baseline and Follow‐Up

**FIGURE 2:**
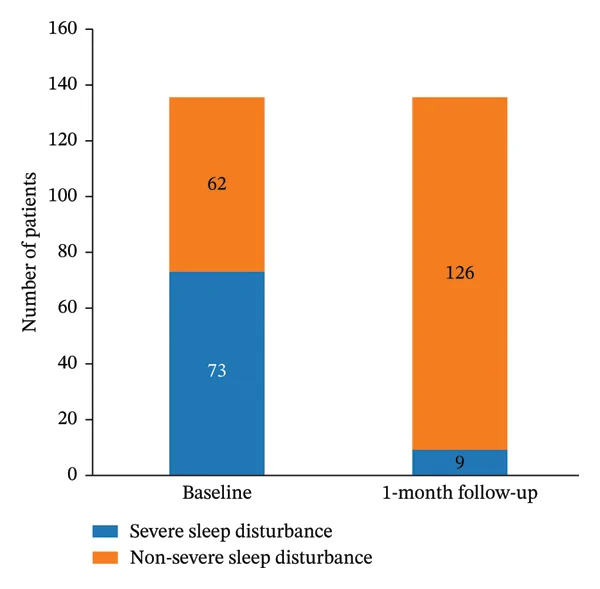
Baseline and 1‐month follow‐up distribution of severe and nonsevere sleep‐disturbance status.

Using the prespecified severity‐based threshold of PSQI > 10, 73 patients had severe sleep disturbance at baseline, whereas 9 patients had severe sleep disturbance at the 1‐month follow‐up. McNemar’s test showed a significant within‐subject change in severe sleep‐disturbance status (*p* < 0.001). Because the study lacked a control group, this change should be interpreted as a within‐subject temporal change after PRF rather than evidence of a PRF‐specific treatment effect (Figure 2).

### 3.4. SII

**FIGURE 3:**
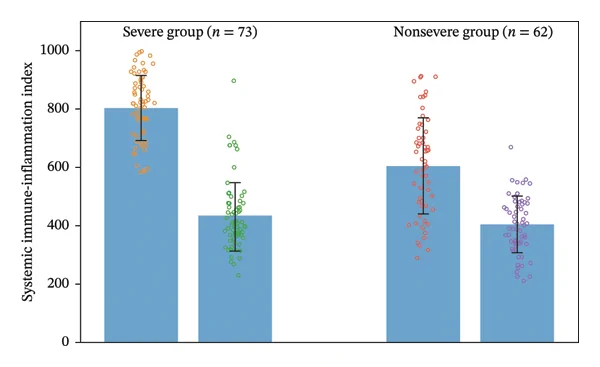
Systemic immune‐inflammation index at baseline and 1‐month follow‐up, stratified by baseline sleep‐disturbance severity. Bars represent mean ± standard deviation, and dots represent individual participants.

At baseline, SII was higher in the severe group than in the nonsevere group (803.03 ± 111.45 vs. 604.21 ± 164.29, *p* < 0.001). At the 1‐month follow‐up, SII decreased in both the severe group (803.03 ± 111.45 to 430.20 ± 117.12, *p* < 0.001) and the nonsevere group (604.21 ± 164.29 to 404.31 ± 97.50, *p* < 0.001). The between‐group difference at follow‐up was not statistically significant using a nonparametric comparison (*p* = 0.498). (Figure 3).

### 3.5. Urinary Melatonin Concentration

**FIGURE 4:**
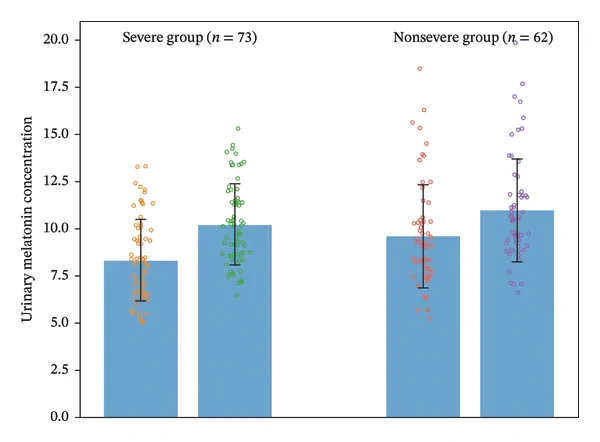
Unadjusted first‐morning spot urinary melatonin concentration at baseline and 1‐month follow‐up, stratified by baseline sleep‐disturbance severity. Bars represent mean ± standard deviation, and dots represent individual participants.

Baseline unadjusted first‐morning spot urinary melatonin concentration was lower in the severe group than in the nonsevere group (8.32 ± 2.17 vs. 9.61 ± 2.72, *p* = 0.006). At the 1‐month follow‐up, urinary melatonin concentration increased in both the severe group (8.32 ± 2.17 to 10.21 ± 2.14, *p* < 0.001) and the nonsevere group (9.61 ± 2.72 to 10.96 ± 2.74, *p* < 0.001). The between‐group difference at follow‐up was not statistically significant (*p* = 0.127). These findings should be interpreted cautiously because urinary creatinine correction was not performed and aMT6 s was not measured (Figure 4).

### 3.6. Availability of Standardized Pain Severity and Medication Data

Standardized longitudinal VAS or NRS pain‐intensity scores were not available in a complete and analyzable format. NRS was used clinically to determine rescue medication after PRF, and gabapentin 100 mg was allowed only when NRS was > 4. However, timing, frequency, cumulative dose, and longitudinal pain trajectories were not systematically recorded for statistical adjustment. Therefore, we could not determine whether the observed temporal changes in SII, urinary melatonin concentration, or severe sleep‐disturbance status were independent of pain relief or rescue medication exposure.

### 3.7. Limited Exploratory Logistic Regression Analysis

A limited exploratory logistic regression analysis was performed using available baseline covariates. In univariate analyses, baseline SII was associated with severe sleep‐disturbance status. In the multivariable model adjusted for sex and BMI, baseline SII remained associated with severe sleep‐disturbance status (adjusted OR per 1‐unit increase: 1.011; 95% CI: 1.007–1.014; *p* < 0.001). This result should be interpreted as a limited exploratory association because several clinically important confounders were unavailable for adjustment (Table 2).

**TABLE 2:** Limited univariate and multivariate logistic regression analyses of available baseline covariates associated with severe sleep‐disturbance status.

	Univariate			Multivariate		
	OR	95% CI	*p*	OR	95% CI	*P*
Gender	0.787	0.399, 1.555	0.491	0.555	0.232, 1.326	0.185
BMI	0.923	0.811, 1.051	0.226	0.854	0.724, 1.006	0.059
SII	1.010	1.007, 1.014	< 0.001	1.011	1.007, 1.014	< 0.001

*Note:* The multivariable model was limited to available baseline covariates and should be interpreted as exploratory. Important variables such as standardized pain‐intensity trajectories, psychological status, sleep apnea status, detailed medication exposure, and fully powered etiology‐specific effects were not included.

### 3.8. Exploratory ROC Analysis

**FIGURE 5:**
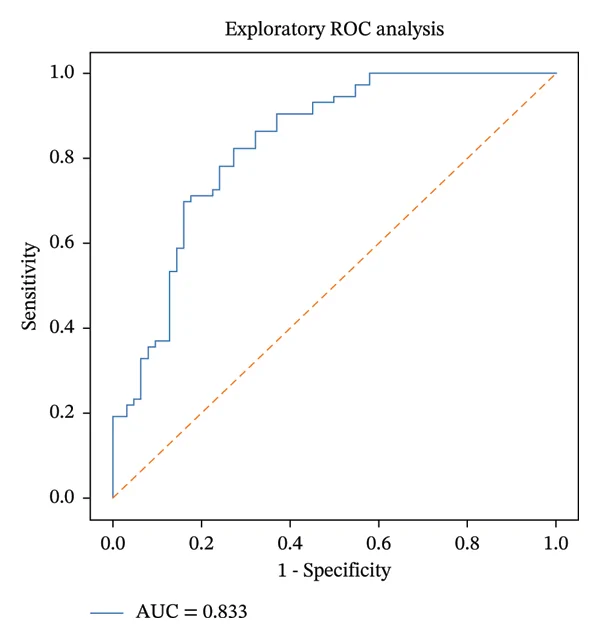
Exploratory ROC curve for baseline SII in discriminating severe sleep‐disturbance status. The AUC and cutoff are apparent in‐sample estimates and require validation.

Exploratory ROC analysis showed an apparent in‐sample AUC of 0.833 (bootstrap 95% CI: 0.757–0.900) for baseline SII in discriminating severe sleep‐disturbance status. The exploratory in‐sample cutoff was 686.68, with sensitivity of 82.2% and specificity of 72.6%. Because no internal or external validation, calibration assessment, or clinical utility analysis was performed, this cutoff should not be considered a validated clinical decision threshold (Figure 5).

## 4. Discussion

In this prospective longitudinal observational cohort of patients with neuropathic pain, higher baseline SII was associated with severe sleep‐disturbance status defined by a prespecified PSQI of > 10 severity threshold. Patients in the severe group also had lower unadjusted first‐morning spot urinary melatonin concentrations at baseline. At 1 month after PRF, SII decreased, urinary melatonin concentration increased, and the number of patients meeting the severe sleep‐disturbance threshold decreased. However, these longitudinal changes should be interpreted as descriptive within‐subject temporal changes rather than PRF‐specific effects because no control group was included.

The finding that baseline SII was associated with severe sleep‐disturbance status is biologically plausible but should not be interpreted causally. SII integrates platelet, neutrophil, and lymphocyte counts and may reflect systemic inflammatory and immune balance [8–10]. Sleep and inflammation are known to interact bidirectionally, and inflammatory cytokines such as IL‐6, TNF, and IL‐1 have been implicated in sleep regulation [15, 16]. Nevertheless, the present study did not measure these cytokines, and the baseline association was derived from variables measured at the same time point. Therefore, the data do not establish whether systemic inflammation contributes to sleep disturbance, whether sleep disturbance increases inflammatory activity, or whether both reflect shared confounding.

The melatonin findings also require cautious interpretation. aMT6 s is generally considered the major urinary metabolite used to estimate nocturnal melatonin production [10, 11]. In this study, native urinary melatonin rather than aMT6 s was measured in a first‐morning spot urine sample. In addition, urinary creatinine was not measured, and creatinine correction was not performed. Hydration status, urine concentration, sample timing, storage conditions, and assay variability may therefore influence the measured values. Accordingly, these results should be interpreted as unadjusted spot urinary melatonin concentrations rather than accurate estimates of total nocturnal melatonin secretion.

A central limitation is the absence of standardized longitudinal pain severity data. Pain intensity is closely related to sleep quality, psychological distress, medication use, and inflammatory status [17–20]. Although NRS was used clinically to guide rescue gabapentin administration after PRF, complete NRS trajectories and rescue‐medication exposure were not available for statistical adjustment. Therefore, the observed changes in SII, urinary melatonin concentration, and severe sleep‐disturbance status may partly or largely reflect pain relief rather than a direct inflammation–melatonin–sleep pathway.

The multivariable regression and ROC analyses should also be interpreted conservatively. The regression model was limited to available baseline covariates and could not include several important determinants of sleep quality, including standardized pain severity, depression/anxiety symptoms, psychiatric comorbidities, sleep apnea screening, detailed medication exposure, and adequately powered etiology‐specific effects. Thus, residual confounding may have overestimated the apparent association between SII and severe sleep‐disturbance status. Similarly, the ROC cutoff was derived from the same single‐center cohort and was not validated; therefore, it should not be used as a clinical decision threshold without further validation.

The mixed neuropathic pain cohort also limits interpretability. Trigeminal neuralgia, herpes zoster–related neuralgia, intercostal neuralgia, peripheral neuropathy, headache‐related neuropathic pain, and lumbar disc–related radicular pain may differ in pathophysiology, inflammatory burden, pain severity, sleep‐disruption patterns, and treatment response. Although disease duration was restricted to 6–12 months to reduce heterogeneity related to chronicity, etiological heterogeneity remained substantial. Future studies should focus on more homogeneous neuropathic pain populations or recruit larger samples that allow prespecified subgroup analyses and interaction testing.

This study should therefore be viewed as exploratory and hypothesis‐generating. Its strength is that it prospectively collected repeated inflammatory and urinary melatonin–related measurements in a clinically relevant neuropathic pain cohort. However, the lack of a control group, incomplete adjustment for key confounders, limited pain and medication data, urinary melatonin assay limitations, absence of cytokine measurements, and lack of ROC validation substantially limit causal and clinical interpretation.

## 5. Conclusion

In this mixed neuropathic pain cohort, higher baseline SII was associated with severe sleep‐disturbance status, and lower unadjusted first‐morning spot urinary melatonin concentration was observed in patients with severe sleep disturbance. At 1 month after PRF, within‐subject decreases in SII, increases in urinary melatonin concentration, and reductions in severe sleep‐disturbance status were observed. However, these findings do not establish causality, PRF‐specific efficacy, a direct inflammation–melatonin–sleep mechanism, or a validated predictive cutoff. Larger controlled studies with standardized pain‐intensity assessment, detailed medication recording, psychological and sleep‐apnea screening, cytokine profiling, validated circadian/melatonin measures, and internal and external validation are needed.

## Author Contributions

Jialei Zhang and Jie Wu designed the study and wrote the manuscript. Jialei Zhang, Jing Li, Kai Cao, and Xiaoling Zhang performed the experiments and analyzed the data. Jialei Zhang and Xiaoling Zhang wrote this paper. All authors contributed to the article.

## Funding

The authors declare that no financial support was received for the research, authorship, and/or publication of this article.

## Disclosure

All authors approved the submitted version.

## Ethics Statement

The experimental protocol was established according to the ethical guidelines of the Helsinki Declaration and was approved by Changzhi People’s Hospital Medical Ethics Committee. Written informed consent was obtained from individual or guardian participants.

## Consent

Please see the Ethics Statement.

## Conflicts of Interest

The authors declare no conflicts of interest.

## Data Availability

The data that support the findings of this study are available on request from the corresponding author. The data are not publicly available due to privacy or ethical restrictions.
